# A Modified MELD Model for Chinese Pre-ACLF and ACLF Patients and It Reveals Poor Prognosis in Pre-ACLF Patients

**DOI:** 10.1371/journal.pone.0064379

**Published:** 2013-06-05

**Authors:** Qi Xia, Xiahong Dai, Yimin Zhang, Yongzheng Guo, Xiaowei Xu, Qian Yang, Weibo Du, Xiaoli Liu, Yuemei Chen, Jianrong Huang, Lanjuan Li

**Affiliations:** State Key Laboratory for Diagnosis and Treatment of Infectious Diseases, The First Affiliated Hospital, School of Medicine, Zhejiang University, Hangzhou, Zhejiang, China; The University of Hong Kong, Hong Kong

## Abstract

**Background & Aims:**

Acute-on-chronic liver failure (ACLF) is one of the most deadly, prevalent, and costly diseases in Asia. However, no prognostic model has been developed that is based specifically on data gathered from Asian patients with ACLF. The aim of the present study was to quantify the survival time of ACLF among Asians and to develop a prognostic model to estimate the probability of death related to ACLF.

**Methods:**

We conducted a retrospective observational cohort study to analyze clinical data from 857 patients with ACLF/pre-ACLF who did not undergo liver transplantation. Kaplan–Meier and Cox proportional hazards regression model were used to estimate survival rates and survival affected factors. The area under the receiver operating characteristic curve (auROC) was used to evaluate the performance of the models for predicting early mortality.

**Results:**

The mortality rates among patients with pre-ACLF at 12 weeks and 24 weeks after diagnosis were 30.5% and 33.2%, respectively. The mortality rates among patients with early-stage ACLF at 12 weeks and 24 weeks after diagnosis were 33.9% and 37.1%, respectively. The difference in survival between pre-ACLF patients and patients in the early stage of ACLF was not statistically significant. The prognostic model identified 5 independent factors significantly associated with survival among patients with ACLF and pre-ACLF: the model for end-stage liver disease (MELD) score; age, hepatic encephalopathy; triglyceride level and platelet count.

**Conclusion:**

The findings of the present study suggest that the Chinese diagnostic criteria of ACLF might be broadened, thus enabling implementation of a novel model to predict ACLF-related death after comprehensive medical treatment.

## Introduction

Acute-on-chronic liver failure (ACLF) is a life-threatening clinical syndrome with a complicated etiology, varied manifestations and a short-term mortality rate of 50–90% [1]. Although liver transplant is considered to be the most effective treatment for ACLF, few patients benefit from this approach because of the shortage of liver donors and the high cost of the procedure [2]. Thus, comprehensive medical intervention which included absolute bed rest, energy supplements, intravenous drop infusion of albumin or plasma, maintenance of electrolyte or acid-base equilibrium, the use of glutathione, adenosylmethionine or branched-chain amino acids to nourish the liver cells, prevention and treatment for complications, is still the mainstay of treatment for patients with ACLF. In such cases, both early diagnosis of ACLF and early prediction of prognosis are critical for distinguishing patients who require transplantation from those individuals who will survive following only intensive medical care.

Currently, the most commonly used models to assess the severity of liver disease include the Child–Turcotte–Pugh (CTP) scoring system and the model for end-stage liver disease (MELD) scoring system [3]–[4]. CTP was established as early indicator for bleeding oesophageal varices. The MELD scoring system was established to evaluate the short-term prognosis of patients with liver cirrhosis after TIPS. It was used as a liver transplant liver source allocation basis to determine the order of patients undergoing liver transplantation by the United Network for Organ Sharing (UNOS). MELD-Na is modified MELD which introduced serum sodium concentration^5^. These models may have several drawbacks when used to determine survival among Asian patients with ACLF. First, ACLF was defined as the acute deterioration of preexisting chronic liver disease with or without cirrhosis; however, the existing models were not established using data from patients with ACLF. The CTP, the MELD and the MELD-Na scoring systems were originally established to evaluate the short-term mortality risk among patients with cirrhosis [3]–[5]. Second, all these existing models were established among European and American population. And the etiology of ACLF differs by geographic location. The causes of ACLF among European and American patients are typically alcohol consumption, hepatitis C and cholestasis. However, among Asian patients, the causes are various. The commonest cause is hepatitis B, while additional causes usually exist, such as hepatitis B plus alcohol, hepatitis B plus C, autoimmune hepatitis plus virus hepatitis. The existing ACLF prognosis models (logistic regression model, LRM and Maddrey score) were used to analyze HBV-induced or alcohol-induced ACLF only and may not be suitable for clinical practice owing to the varied causes of ACLF [1], [6]–[7] among Asian patients. Third, these models were established with untreated patients. In other words, these models considered prognosis related to the natural history of the underlying condition, but did not consider the impact of medical treatment on the prognosis. So, it is necessary to establish a new prognostic model based on Asian patient with ACLF after comprehensive medical treatment.

Furthermore, in our clinical practice we have observed poor prognoses among some pre-ACLF patients whose serum total bilirubin and/or international normalized ratio (INR) for prothrombin time did not meet the Chinese ACLF diagnosis criteria (5 mg/dL ≤ serum total bilirubin <10 mg/dL and 1.28≤ INR <1.50 or 40% <PTA ≤60%). Therefore, pre-ACLF patients were also included in the analysis.

The aim of the present study was to assess the survival time of ACLF among Chinese individuals and to develop a prognostic model for use in this population.

## Patients and Methods

### Ethics Statement

The research protocol was approved by the Human Ethics Committee of the First Affiliated Hospital, School of Medicine, Zhejiang University, and all enrolled participants signed a written informed consent to participate in the study. Data was entered in duplicate into a computerized database and was analyzed anonymously.

### Study Design

A retrospective observational study of factors associated with survival among patients with ACLF was conducted. Eligible patients had attended the First Affiliated Hospital, School of Medicine, Zhejiang University during the period 1 December 2008 to 1 February 2012. The research was performed according to Standards of the Reporting of Diagnostic Accuracy Studies [8].

### Disease Definition

The criteria for the Chinese definition of ACLF were in accordance with the “Guideline for Diagnosis and Treatment of Liver Failure” [9]. The ACLF criteria included the following four features: (1) acute deterioration of preexisting chronic liver disease; (2) extreme fatigue with severe digestive symptoms, such as obvious anorexia, abdominal distension, nausea and vomiting; (3) progressively worsening jaundice within a short period (serum total bilirubin level ≥10 mg/dL or a daily elevation ≥1 mg/dL); (4) an obvious hemorrhagic tendency with prothrombin activity [10](PTA) ≤40% (approximate prothrombin time, PT ≥18.3 s, international normalized ratio, INR >1.50). Any lack of the above four was insufficient to diagnose ACLF. As shown in Table 1, patients with ACLF were subdivided into early, intermediate and late stages of disease according to the criteria list above [9], [11].

**Table 1:** Chinese staging criteria for acute-to-chronic liver failure.

Parameter	Early stage	Intermediate stage	Late stage
Total serum bilirubin (mg/dL)	≥10 or ≥1 per day	≥10 or ≥1 per day	≥10 or ≥1 per day
PTA (%)	≤40 and >30	≤30 and >20	≤20
HE and/or ascites	No	HE (≤ grade II) and/or obvious ascites	HE (≥ grade III)
Complications	No	No	UGIB, HRS, severe infections, ascites

Abbreviations: HE, hepatic encephalopathy; HRS hepatorenal syndrome; PTA, prothrombin time activity; UGIB upper gastrointestinal bleeding.

We defined patients whose serum total bilirubin level and/or INR for prothrombin time did not meet the Chinese ACLF criteria as having “pre-ACLF.” Our definition for Chinese pre-ACLF included the following features: (1) acute worsening of chronic liver disease; (2) extreme fatigue with severe digestive symptoms, such as obvious anorexia, abdominal distension, nausea and vomiting; (3) progressively worsening jaundice within a short period, with 5 mg/dL ≤ serum total bilirubin level <10 mg/dL or a daily elevation of ≥1 mg/dL; (4) 40% <PTA ≤60% or 1.28≤ INR <1.50.

Hepatic encephalopathy (HE) was defined as neuropsychiatric abnormalities during the course of liver disease, including involvement of the cognitive, affective/emotional, behavioral, and bioregulatory domains. Clinical grading of the abnormal mental state was used for quantification [11]. Clinical grading of the abnormal mental state was used for quantification as stages I–IV [11]–[12].

1. Stage I: Loss of sleep rhythm, drowsiness, confusion and flapping tremors.
2. Stage II: Features of grade I encephalopathy with loss of sphincter control as well.
3. Stage III: Unconsciousness with no response to oral commands, but responding to painful stimuli.
4. Stage IV: Deep unconscious state, with no response to pain.

The CTP classification was assessed according to standard criteria^3^, and MELD scores were calculated according to the Malinchoc formula [4]:

R = 9.57*log_e_(creatinine[mg/dL])+3.78*log_e_(bilirubin[mg/dL])+11.2*log_e_(INR)+6.43*(etiology: 0 if cholestatic or alcoholic, 1 otherwise).

MELD-Na score was calculated according to the following formula [5]:

MELD-Na = MELD+1.59*(135-serum sodium)^.^

### Study Population, Follow-up, and Data Collection

**Figure 1:**
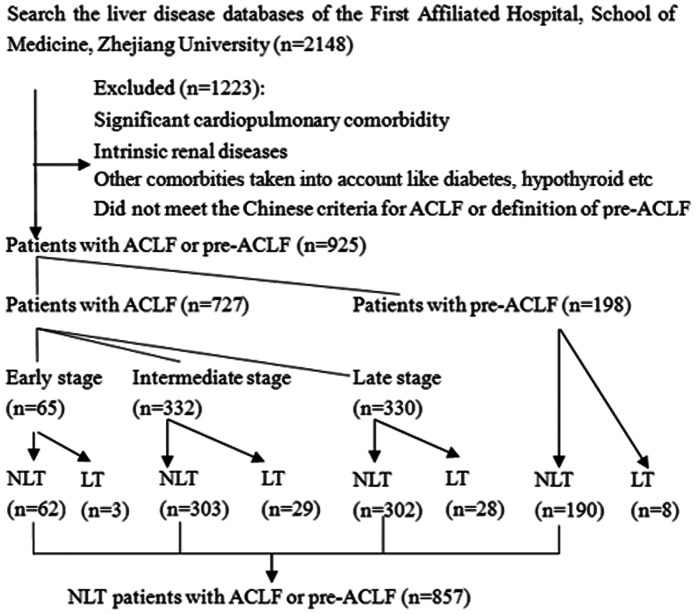
Flowchart of the study group selection process. LT: patients underwent liver transplantation; NLT: patients without liver transplantation.

The flow chart of the study group selection process is presented in Figure 1. The liver disease databases of the Hospital were searched using the following query terms: * liver failure * or *severe hepatitis* or *severe hepatology* or *cirrhosis and decompensation* or *decompensated cirrhosis* (* here means a wildcard in searches). In total, 2148 cases of suspected liver failure were found between 1 December 2008 and 1 February 2012. Of these, 1291 patients were excluded because they had marked cardiopulmonary comorbidity or intrinsic renal disease or did not meet the defined ACLF or pre-ACLF criteria. The remaining 925 patients fulfilled the Chinese criteria for ACLF and pre-ACLF; the 857 patients who had not undergone liver transplantation were included in the final analysis. The 857 patients received comprehensive medical intervention which included absolute bed rest, energy supplements, intravenous drop infusion of albumin or plasma, maintenance of electrolyte or acid-base equilibrium, the use of glutathione, adenosylmethionine or branched-chain amino acids to nourish the liver cells, prevention and treatment for complications. Oral antiviral treatment including Lamivudine, Adefovir Dipivoxil, Telbivudine and Entecavir were ordered to the patients in whom hepatitis B virus activated replication.

The factors predicting survival after comprehensive medical treatment were studied in the patients with ACLF or pre-ACLF. We examined the medical records of the 857 patients who fulfilled the Chinese criteria for ACLF or pre-ACLF from their date of admission until either their death or the closure of the study (May 31, 2012).

Descriptive statistics on the patients’ features were recorded within 24 hours of the diagnosis date. The prothrombin time was converted to prothrombin time activity percentage (PTA). The prothrombin time in seconds was converted to the international normalized ratio (INR) using the international sensitivity index (ISI) for thromboplastin [10]. Because of the specimen collection variation within 24 hours of the date of diagnosis, data on one of the following parameters was lacking for 23 of the patients: serum bilirubin level, albumin concentration, sodium concentration, INR, or triglyceride level recorded within 24 hours of the date of admission. Missing values were automatically deleted by SPSS software version 16.0 (SPSS, Chicago, IL).

### Kaplan-Meier Analysis

The actual survival of patients with pre-ACLF, early-stage ACLF, intermediate-stage ACLF and late-stage-ACLF were compared by Kaplan-Meier analysis [13]. Comparison of actual (Kaplan-Meier) survival for patients with different primary causes, such as HBV alone, HBV combined with alcohol, alcohol alone or other primary causes were performed. Other primary causes included HBV combined with schistosome; HCV; alcohol combined with schistosome; autoimmune and cholestatic. However, the results showed revealed interference caused by confounding factors. Similarly, comparisons of actual survival were also analyzed according to the acute causes. The acute causes were divided into four groups as HBV alone, Hepatitis B plus other cause, drug and others. Hepatitis B plus other cause included HBV combined with drugs, HBV combined with alcohol, HBV combined with HGV, HBV combined with HAV, and HBV combined with surgery/trauma. Other acute causes included HGV, alcohol, HCV, autoimmune, surgery/trauma, and cholestatic.

### Survival Modeling

The start time for all survival analysis was the date of diagnosis. The patients who were lost to follow-up were censored at the date that they were last known to be alive. The term ‘censored’ indicates that the patient was alive at that date and that was the extent of the follow-up [14].

The patients who met Chinese diagnostic criteria of ACLF and pre-ACLF were divided into the training cohort and the validation cohort. The patients who were admitted to the hospital from Dec 1, 2008 to Sep 30, 2011 were included in the training cohort (*n* = 758). Those who were admitted to the hospital from Feb 1, 2011 to May 31, 2012 were included in the validation cohort (*n* = 99).

For the training cohort, bivariate analysis was performed for determining the association of the clinical and laboratory parameters with the prognosis. The candidate variables which were statistically significant (*P*<0.0001) in bivariate assessment of risk factors for death were named the candidate variables. These candidate variables were included in a forward-conditional stepwise Cox proportional hazards regression [13]–[15] to identify independent predictors for the prognosis of the patients with ACLF and pre-ACLF. For this analysis, the conditional probabilities for stepwise entry and removal of a factor were 0.05 and 0.10, respectively. To lessen the influence of extreme laboratory values, we transformed the quantitative variables to their natural logarithms.

### Model Validation

In the validation cohort, the actual survival was calculated using the Kaplan-Meier procedure [13], and the predicted survivals were compared using a one sample log rank test [15]. A P value below 0.05 was used to indicate significant differences between the observed and predicted survival. Furthermore, the standard indices of validity, such as sensitivity, specificity, positive predictive value, negative predictive value, positive likelihood ratio, negative likelihood ratio and area under the receiver operating curve (auROC), which is a measure of discrimination (*i.e.* the ability of the model to distinguish patients who survive from those who do not) were calculated.

Statistical analysis were performed using SPSS software version 16.0. Continuous values were expressed as the mean ± SD or as the median and the interquartile range, and categorical values were described by counts and proportions. For all analysis, a P value of less than 0.05 was considered statistically significant.

## Results

### Patient Demographics and Follow-up

As shown in Figure 1, in total 925 patients with ACLF or pre-ACLF were included in analysis. In all, 60 patients with ACLF underwent orthotopic liver transplantation, while 667 patients did not. Similarly, 8 patients who fulfilled the definition of pre-ACLF underwent orthotopic liver transplantation, while 90 patients did not. The ACLF patients were divided into groups based on the early, intermediate and late stages of disease [9]. As survival among ACLF patients who underwent orthotopic liver transplantation (OLT) was significantly improved compared with those who did not undergo OLT, the 68 OLT patients were removed when modeling the survival of ACLF patients who underwent comprehensive medical treatment (Figure 1). The baseline characteristics of patients with ACLF or pre-ACLF who did not undergo OLT (*n* = 857) were indicated in Table S1. Ascites were present in 84.8% of the patients and we scored this using three degrees of severity. Some degree of hepatic encephalopathy was observed in 31.9% of the patients and was staged using standard criteria I–IV. In 857 patients, the mean CTP score was 10±1. The mean MELD score was 2.35±0.90, and the mean MELD-Na score was 2.76±4.88. The primary cause of liver disease was hepatitis B (70.3%), hepatitis B combined with alcohol abuse (17.4%) or alcohol abuse (6.5%), with the remaining patients (5.8%) having other types of liver disease. The acute cause of liver disease was hepatitis B (55.3%), hepatitis B combined with another cause (29.5%) or drug-related (1.1%), with the remaining patients (14.1%) having another acute cause.

The 857 patients who fulfilled the Chinese criteria for ACLF or pre-ACLF were followed from their date of admission until either their death or the closure of the study (May 31, 2012). As shown in Table 2, 501 patients died during follow-up and 63 were lost to follow-up; the remaining 293 patients were still alive at study end. In detail, 470 patients died of liver failure within 12 weeks, and 485 died within 24 weeks. The deaths were related to complications from liver disease, as hepatic encephalopathy (HE), hepatorenal syndrome (HRS), gastrointestinal bleeding, sever infection and hepatopulmonary syndrome. Of the 857 patients included in the analysis, 407 had less than 12 weeks’ follow-up and 499 patients had less than 24 weeks’ follow-up. The average duration of follow-up was 7 weeks (range 0.0 to 183.1 weeks).

**Table 2:** Follow-up data for the patients who did not undergo liver transplantation.

	All patients(*n* = 857)	pre-ACLF(*n* = 190)	Early-stageACLF (*n* = 62)	Intermediate-stageACLF (*n* = 303)	Late-stageACLF (*n* = 302)
Mean follow-up (weeks) *	7.0	57.0	60.6	10.4	1.6
Range of follow-up (weeks)	0.0–183.1	0.1–174.5	0.9–177.0	0.1–181.9	0.0–183.1
Deaths (*n*)	501	66	23	170	242
Dates of hospital admission					
First	11/26/08	12/24/08	01/08/09	12/03/08	11/26/08
Last	02/09/12	01/26/12	02/09/12	02/07/12	02/01/12

Abbreviation: ACLF, acute-to-chronic liver failure.

* Among the 356 patients known to be alive at the last follow-up.

### Patient Survival

**Figure 2:**
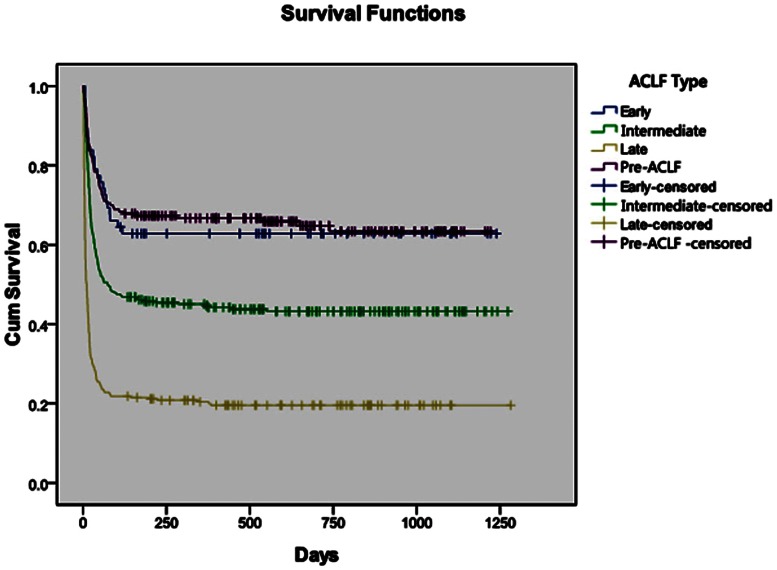
Comparison of actual survival among patients with pre-ACLF, early-stage ACLF, intermediate-stage ACLF and late-stage ACLF. No significant differences existed between patients with pre-ACLF and early-stage ACLF (P>0.05). However, the difference in survival between pre-ACLF patients and intermediate-stage ACLF patients was statistically significant (P<0.0001). The difference in survival between intermediate-stage ACLF patients and late-stage ACLF patients was also statistically significant (P<0.0001).

The actual survival of patients with pre-ACLF, early-stage ACLF, intermediate-stage ACLF and late-stage ACLF were compared by Kaplan-Meier analysis. The median survival time of the 857 patients with ACLF and pre-ACLF was 7 weeks. The 12-week and 24-week survival rates were 54.8% and 58.2%, respectively. In detail, approximately 30.5% of the pre-ACLF patients died of liver failure within 12 weeks, and 33.2% died within 24 weeks after diagnosis. Approximately 33.9% of the early-stage ACLF patients died of liver failure within 12 weeks, and 37.1% died within 24 weeks. Approximately 49.5% of the intermediate-stage ACLF patients died of liver failure within 12 weeks, and 53.8% died within 24 weeks. Approximately 77.2% of the late-stage ACLF patients died of liver failure within 12 weeks, and 78.5% died within 24 weeks. The difference in survival between the pre-ACLF patients and the early-stage ACLF patients was not statistically significant (*P*>0.05). The mortality rates of the pre-ACLF patients within 12 weeks and 24 weeks were lower than the mortality rates of the intermediate-stage ACLF patients. The difference in survival between the pre-ACLF patients and the intermediate-stage ACLF patients was statistically significant (*P*<0.0001). The mortality rates of the intermediate-stage ACLF patients within 12 weeks and 24 weeks were lower than the mortality rates of the late-stage ACLF patients. The difference in survival between the intermediate- and late-stage ACLF patients was statistically significant (*P*<0.0001). (Fig. 2).

**Figure 3:**
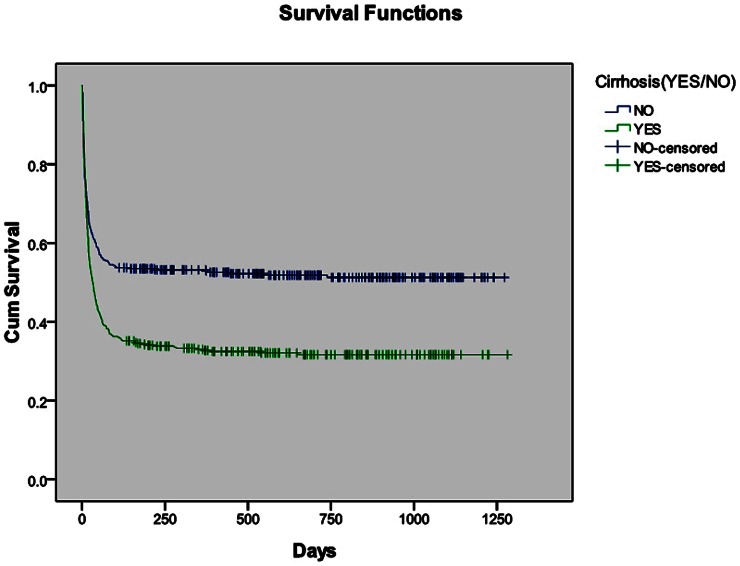
Survival comparison of patients with and without cirrhosis. The difference in survival between patients with cirrhosis and without cirrhosis was statistically significant (P<0.0001).

The actual survival of all patients with cirrhosis (*n* = 455) was compared to that of patients without cirrhosis (*n* = 402). The mortality of patients with cirrhosis was 63.1% within 12 weeks and 65.5% within 24 weeks. The mortality of patients without cirrhosis was 45.5% within 12 weeks and 46.5% within 24 weeks. Figure 3 indicates that the patients with cirrhosis had a 17.6% greater risk of dying by 12 weeks compared to the patients without cirrhosis. The difference in survival between the above two groups was statistically significant (*P*<0.0001). Similarly, patients with cirrhosis had a 19% greater risk of dying by 24 weeks when compared to patients without cirrhosis. The difference in survival between patients with cirrhosis and without cirrhosis was statistically significant (*P*<0.0001).

### Bivariate Analysis

The patients with ACLF and pre-ACLF were divided into a training cohort and a validation cohort. Patients who were admitted to the hospital from Dec 1, 2008 to Sep 30, 2011 were included in the training cohort (*n* = 758). Those who were admitted to the hospital from Feb 1, 2011 to May 31, 2012 were included in the validation cohort (*n* = 99). The demographic, clinical, biochemical and hepatic-hemodynamic features in the training and validation cohorts were described in Table S2.

For the training cohort (*n* = 758), the candidate variables were those factors shown in Table 3 that were statistically significant (*P*<0.0001) in a bivariate assessment of risk factors for death. As displayed, those factors increasing age, cirrhosis, hepatic encephalopathy and MELD score significantly increased the risk of death, whereas increasing levels of serum albumin, platelets, triglycerides and sodium indicated a significantly decreased the risk of death.

**Table 3:** Bivariate assessment of risk factors for death by any cause among the training cohort.

Variable	Patients (n)	Regression coefficient	Regression coefficient standard error	*p* value
**Demographic**				
Age	758	0.029	0.004	<0.0001
**Clinical**				
Cirrhosis	758	0.405	0.097	<0.0001
Hepatic encephalophy‡	758	0.399	0.039	<0.0001
**Biochemical**				
Albumin (loge value)	752	-1.718	0.317	<0.0001
Platelets (10^9^/L) (loge value)	758	-0.589	0.075	<0.0001
Sodium (mEq/L)(loge value)	757	-4.704	1.150	<0.0001
Triglycerides (log e value)	745	-0.560	0.072	<0.0001
**Scoring system**				
MELD	758	0.681	0.042	<0.0001

All analysis were performed by the Cox proportional hazard regression. Entries are the regression coefficients and their standard errors from a bivariate Cox proportional hazards regression model. Positive coefficients imply that the risk of death increases with increasing values of a risk factor. The P values reflect the role of chance in these findings (that is, the test the regression coefficient = 0). The relative risk attributable to a risk factor can be estimated from the regression coefficients. ‡ Coded: 0 = stage 0 to 4 = stage IV.

### Multivariate Analysis

Of the candidate variables derived from the bivariate analysis, only the MELD score, age, hepatic encephalopathy, log_e_ platelet count and log_e_ triglyceride level were independently predictive of survival (Table 4). These variables were predictive of survival for both the ACLF and pre-ACLF patients after comprehensive medical treatment in the multivariate analysis (Table 5). A 100% increase in the MELD score multiplied the risk of death by 1.665 times; a 100% increase in age multiplied the risk of death by 1.022 times; a 100% increase in hepatic encephalopathy multiplied the risk of death by 1.322 times, a 100% decrease in platelet count multiplied the risk of death by 0.811 times; and a 100% decrease in triglyceride levels multiplied the risk of death by 0.838 times. Table 5 presents the underlying survival function, *i.e.* the survival of a patient with ACLF or pre-ACLF with a risk score of 2.501(the mean risk score of the 758 patients from the training cohort).

**Table 4:** Survival model for the training cohort*.

Variable	Regression coefficient	Regression coefficient standard error	P value
Age	0.021	0.004	<0.0001
Hepatic encephalophy	0.279	0.044	<0.0001
Platelets (log_e_ value)	-0.210	0.087	0.016
Triglycerides (log_e_ value)	-0.176	0.074	0.017
MELD score	0.513	0.049	<0.0001

* *n* = 758; deaths = 447.

**Table 5:** Candidate variables for survival predicting through multivariate analysis.

Characteristic	Multivariate analysis		
	RR	95% CI	P value
MELD score	1.671	1.518–1.839	p<0.001
Age	1.022	1.014–1.029	p<0.0001
Hepatic encephalopathy	1.322	1.212–1.442	p<0.0001
Triglyceride level	0.838	0.725–0.969	p<0.05
Platelet count	0.811	0.684–0.961	p<0.05

Abbreviations: RR, relative risk; 95% CI, 95% confidence interval; MELD, Model for End-Stage Liver Disease.

### Calculating the ACLF and Pre-ACLF Risk Score and Predicted Survival Probabilities

The risk scores (R) for individual patients can be calculated by combining their 5 prognostic values with the regression coefficients reported in Table 4. That is, R = 0.021×(age in years)+0.279×(hepatic encephalopathy score)+0.513×(MELD score)−0.210×log_e (_platelet count 10^9^/L)−0.176×log_e_ (triglyceride levels mg/dL). For example, a hypothetical patient with hepatitis B who is 50 years old and has an HE of lll, a MELD score of 3.36, a platelet count of 50*10^9^/L and a triglyceride concentration of 80 mg/dL, the risk score would be as follows: R = 0.021×50+0.279×3+0.513×3.36−0.210×log_e_50−0.176×log_e_80 = 2.003.

To obtain the probability of survival for at least t days, one calculates the risk score R, reads S_0_(t) from Table 6, and computes S(t) using the equation S(t) = S_0_(t)^exp(R-R^_0_^)^. R_0_ is the risk score of the average patient in the series and was calculated to be 2.501. For example, for the hypothetical patient with hepatitis B discussed above, the probability of surviving at least 90 days is 0.457^exp(2.003–2.501)^ = 0.62.

**Table 6:** Underlying survival function for the new ACLF prognostic model.

t(days)	1	7	30	90	183	365	730
S_0_(t)*	0.989	0.837	0.594	0.457	0.434	0.422	0.402

* S_0_(t) gives the estimated survival probability for the average ACLF or pre-ACLF patient (risk score 2.501). To calculate the survival of any given patient, the following equation is used: S (t) = S_0_(t)^exp(R-2.501)^. R is calculated from the model provided in Table 4.

### Model Validation and Stability

**Figure 4:**
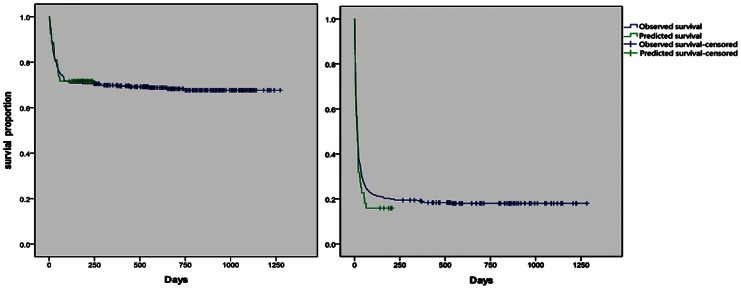
Survival of 97 independent patients with ACLF or pre-ACLF from the validation cohort who were stratified according to their risk score as either low risk (n = 53; left panel) or high risk (n = 44; right panel). The median survival in the high-risk group was less than 12 weeks (R>2.37). In the low-risk group, the median predicted survival was more than 12 weeks (R<2.37). Actual (Kaplan–Meier) and expected survival using the new prognostic model were compared by the one sample log rank test. For the low-risk and high-risk patients, the observed and expected survival rates were similar (P = 0.898 and P = 0.442, respectively).

**Figure 5:**
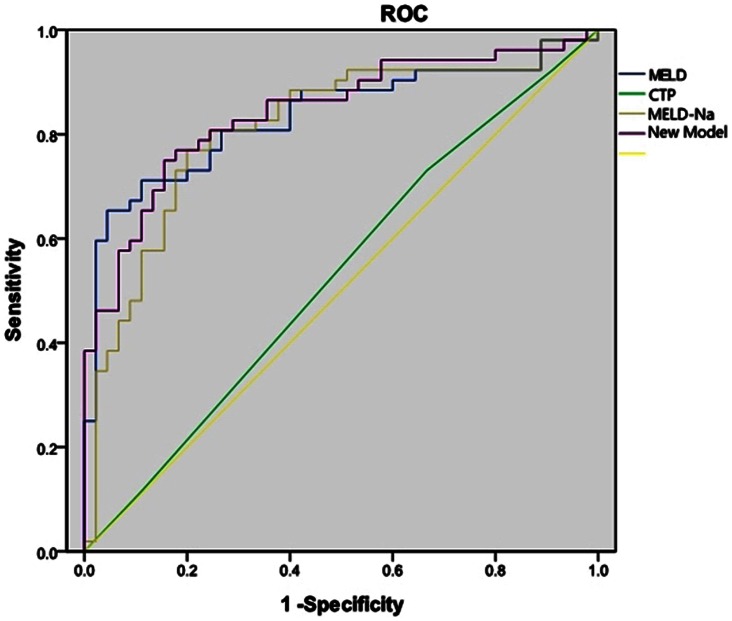
ROC curves of the new model versus CTP, the original MELD scoring system, and MELD-Na scoring systems as predictors of 12 weeks mortality in the validation cohort. The area under ROC curve (95% CI) of the new model, CTP, MELD and MELD-Na scoring systems were 0.84 (0.76–0.92), 0.53 (0.41–0.65), 0.83 (0.75–0.91) and 0.81 (0.72–0.90) respectively.

The actual survival of the validation cohort, which was calculated by the Kaplan-Meier procedure, was compared to the predicted survival using a one-sample log rank test. No significant difference was observed between the two cohorts (Fig. 4). Furthermore, an auROC analysis was performed to test the robustness of the prognostic model. The auROC curve was 0.842 (95% CI 0.763–0.921), and an auROC greater than 50% suggests that the model has utility (Fig. 5). The area under ROC curve (95% CI) of CTP, MELD and MELD-Na scoring systems were 0.53 (0.41–0.65), 0.83 (0.75–0.91) and 0.81 (0.72–0.90) respectively. The AUROC of the new model is significantly different with the AUROC of CTP(p<0.05). While no stastical differents were obtained between the new model and MELD, MELD-Na (p>0.05), which might due to the relatively small sample size.

In the new Chinese-specific model, using a cutoff of 2.2555, the sensitivity was 80.77%, the specificity was 75.56%, the positive predictive value was 79.2, and the negative predictive value was 79.5., the negative likelihood ratio and positive likelihood ratio were 0.25 and 3.30 respectively.

### Comparison with Other Scores

An auROC curve analysis was performed to compare the new model with original prognostic models, such as CTP, MELD and MELD-Na. Figure 5 indicates that the auROC for the new model was 31.3% greater than that for CTP, 0.7% greater than that for MELD and 3% greater than that for MELD-Na.

## Discussion

The definition of pre-ACLF used in this study was proposed on the basis of clinical practice. No statistically significant difference in survival was observed between individuals with pre-ACLF and patients with early-stage ACLF. This observation suggests that patients with pre-ACLF should receive the standard medical therapy for ACLF as soon as possible after diagnosis. Furthermore, on the basis of these results, it is recommended that the original Chinese diagnostic criteria of ACLF might be broadened, while the original stages of ACLF are discarded altogether. Hence, the new ACLF criteria would include the following points: acute deterioration of preexisting, chronic liver disease; extreme fatigue with severe digestive symptoms such as obvious anorexia, abdominal distension, nausea and vomiting; progressively worsening jaundice within a short period, with a serum total bilirubin level ≥5 mg/dL, or a daily elevation ≥1 mg/dL; and PTA ≤60% or INR ≥1.28.

Five independent factors were found to be associated with survival among patients with ACLF and pre-ACLF; namely, MELD score, age, hepatic encephalopathy, triglyceride level and platelet count.

The MELD scoring system was formulated to predict the 3-month mortality among patients with cirrhosis undergoing transjugular intrahepatic portosystemic shunts by Malinchoc in 2000 [4]. The MELD indicators include bilirubin, creatinine, and the INR for prothrombin time, which are objective and tend to be stable. The findings of the present study confirm the prognostic value of the MELD score in predicting short-term mortality among Asian patients with ACLF and pre-ACLF patients after comprehensive medical treatment. The role of the MELD score as a predictor of mortality has already been emphasized by others [16]–[18].

Age was associated with the risk of mortality as a continuous variable, with older patients exhibiting worse survival rates. The result was similar in other reports [6], [16]–[17], [19]–[20] ^6,16.17,19, 20^.

Hepatic encephalopathy is diagnosed by physical examination and considered to be a subjective variable in scoring. Some studies have indicated that hepatic encephalopathy and ascites correlate significantly with mortality in liver failure, particularly among patients with low MELD scores [21]–[22]. Thus, these clinical events should be considered. In the present study, no statistically significant differences were found in survival between the pre-ACLF and early-stage ACLF groups, even if the MELD score was lower among individuals with pre-ACLF than patients with early-stage ACLF. The high mortality of the pre-ACLF group might be associated with hepatic encephalopathy, because some individuals with pre-ACLF had hepatic encephalopathy while the patients with early-stage ACLF did not. Moreover, hepatic encephalopathy was an independent prognostic factor for survival in Cox proportional hazards regression models. Therefore, hepatic encephalopathy was the only subjective indicator integrated into our new prognostic model.

In addition to the lack of indicators for complications (such as hepatic encephalopathy), the MELD score has other drawbacks. Only risk factors and no independent protective factors are included in the MELD score. The new ACLF prognostic model includes triglycerides and platelets as independent prognostic protective factors related to survival. Although it has previously been reported in bivariate assessments that triglycerides and platelets are risk factors for death [6]–[7], [19], [23], this study demonstrates that triglycerides and platelets independently influence survival in addition to the MELD score.

Triglyceride (TG) is composed of long-chain fatty acids and glycerol, which is synthesized in liver. The liver TG is a sensitive indicator to reflect the hepatic reserve functional as it updates quickly. It is commonly used to evaluate the patient's nutritional status as well as cholinesterase and albumin. In the present study, the risk of mortality increased as triglyceride levels decreased. Triglycerides could, therefore, be used not only as a prognostic factor, but also as an observed indicator in a subsequent nutrition therapy study in patients with ACLF. Other studies have revealed that liver nutrition therapy may improve the prognosis [24]–[28]. Moreover, the association of triglycerides with mortality in patients with ACLF will be of interest in subsequent studies.

In the present study, the platelet count was another independent prognostic factors of survival, and the risk of mortality increased as platelets decreased. The decrease of platelets in ACLF may be related to the following mechanisms. First, the decreased syntheses of thrombopoietin lead to production reduce of platelet. Second, bone marrow suppression happened in ACLF, which reduced megakaryocytes proliferation and platelet production. Third, hypersplenism and platelet antibody or complement increased the the destruction of platelet. In addition, DIC in ACLF also increased the consumption of platelet.

Mortality among patients with cirrhosis was significantly higher than that of patients without cirrhosis in the present study. Cirrhosis was a significant variable in the bivariate assessment of risk factors for death. This finding is in accordance with the results of recently published studies on the outcome of cirrhosis that reported cirrhosis as a prognostic indicator of ACLF [6]–[7], [16] ^.^ Surprisingly, however, cirrhosis was not independently predictive of survival variables through multivariate analysis in the present study. The contradiction between these two findings may be related to sample size. It is possible that cirrhosis would be independently predictive of the survival variables in a prognostic model of advanced ACLF in a large group of patients. The relationship between cirrhosis and prognosis suggests that the chronic, severe hepatic damage and the weak regeneration capacity of liver cells resulted in the poor prognosis of patients with ACLF. If the incidence of cirrhosis could be reduced through early diagnosis and treatment, the mortality associated with ACLF might be decreased.

The new prognostic model of ACLF, which we named as Li-ACLF modle, performed better than CTP, MELD-Na and MELD for predicting the 12-week mortality, as shown by the auROC. In the validation sample, the improvement over CTP was 31% (from 0.53 to 0.84). By contrast, the improvement was 3% over MELD-Na (from 0.81 to 0.84) and 1% over MELD (from 0.83 to 0.84). Therefore, these results support the consideration of age, hepatic encephalopathy, triglycerides and platelet count along with the original MELD to improve its accuracy in predicting survival duration.

An ideal survival model should use commonly available determinants, be easily calculated, and be widely applicable to geographically diverse patients. All of the independent factors identified in the present study meet these criteria. However, potential weaknesses in this approach exist. Firstly, this was a retrospective study from a single center. Prospective studies from multiple centers of geographically diverse areas using a larger and more heterogeneous group of patients with a longer-term follow-up evaluation are needed. Secondly, not all of the available published prognosis predication models were compared in the present study. Rather, several of the widely accepted models specific to liver failure were selected. Thirdly, the AUROC of the new model is bigger than the MELD and MELD-Na, but no stastical differents were obtained, which might due to the relatively small sample size. We plan to make further verification in a bigger sample size prospective cohort in a subsequent study.

We intend to validate and optimize the established model in subsequent prospective, multi-center cohort studies. Cirrhosis might be an independent risk factor if the sample size was expanded. We intend to find comprehensive treatment measures to improve the prognosis, including means of preventing triglycerides and platelet declining. We propose a subsequent study to determine the mechanism underlying the decrease in the platelet counts and the prognosis of patients with ACLF, as well as to determine the associated therapy.

Although continued refinement and improvement to the model is anticipated, it may still be useful in its present form for assisting clinicians in the selection of the proper therapy for different groups of patients with ACLF.

## Supporting Information

Table S1 **Baseline characteristics of patients with ACLF and pre-ACLF.** Abbreviations: Abbreviation: ACLF, acute-to-chronic liver failure; INR, international normalized ratio; CTP, Child–Turcotte–Pugh scoring system; MELD, model for end-stage liver disease scoring system Normal distribution continuous values were expressed as the mean±SD. Non-normal distribution continuous values were expressed as the median and interquartile range. *Includes HBV combined with schistosome, HCV, alcohol combined with schistosome, autoimmune and cholestatic. ^#^Includes HGV, alcohol, HCV, autoimmune, surgery/trauma, and cholestatic. ^##^Includes HBV combined with drug, HBV combined with alcohol, HBV combined with HGV, HBV combined with HAV and HBV combined with surgery/trauma. ^$^ Based on hepatic encephalopathy, ascites, bilirubin, albumin, and prothrombin time (seconds). ^&^ Based on bilirubin, creatinine, INR and cause (seconds). ^$$^ Based on bilirubin, creatinine, INR, sodium and cause (seconds). **Comparison of pre-ACLF with early-stage ACLF.
(DOC)

Table S2 **Demographic, clinical, biochemical and hepatic-hemodynamic features in the training and validation cohorts.** Abbreviations: Abbreviation: ACLF, acute-to-chronic liver failure; INR, international normalized ratio; CTP, Child–Turcotte–Pugh scoring system; MELD, model for end-stage liver disease scoring system NOTE: Normal distribution continuous values are expressed as the mean±SD. Non-normal distribution continuous values are expressed as the median and interquartile range. *Includes HBV combined with schistosome; HCV; alcohol combined with schistosome; autoimmune; and cholestatic. ^#^Includes HGV, alcohol, HCV, autoimmune, surgery/trauma, and cholestatic. ^##^Includes HBV combined with drugs, HBV combined with alcohol, HBV combined with HGV, HBV combined with HAV, and HBV combined with surgery/trauma. ^$^ Based on hepatic encephalopathy, ascites, bilirubin, albumin, and prothrombin time (seconds). ^&^ Based on bilirubin, creatinine, INR and cause (seconds). ^$$^Based on bilirubin, creatinine, INR, sodium and cause (seconds). ^**^Comparison of the training cohort with the validation cohort
(DOC)
