# Supplementary material for: A Modified MELD Model for Chinese Pre-ACLF and ACLF Patients and It Reveals Poor Prognosis in Pre-ACLF Patients
Source: PLoS One. 2013 Jun 5;8(6):e64379. doi: 10.1371/journal.pone.0064379 (PMC3673980; doi:10.1371/journal.pone.0064379)
Supplement: Table S1 — Baseline characteristics of patients with ACLF and pre-ACLF. Abbreviations: Abbreviation: ACLF, acute-to-chronic liver failure; INR, international normalized ratio; CTP, Child–Turcotte–Pugh scoring system; MELD, model for end-stage liver disease scoring system Normal distribution continuous values were expressed as the mean±SD. Non-normal distribution continuous values were expressed as the median and interquartile range. *Includes HBV combined with schistosome, HCV, alcohol combined with schistosome, autoimmune and cholestatic. #Includes HGV, alcohol, HCV, autoimmune, surgery/trauma, and cholestatic. ##Includes HBV combined with drug, HBV combined with alcohol, HBV combined with HGV, HBV combined with HAV and HBV combined with surgery/trauma. $ Based on hepatic encephalopathy, ascites, bilirubin, albumin, and prothrombin time (seconds). & Based on bilirubin, creatinine, INR and cause (seconds). $$ Based on bilirubin, creatinine, INR, sodium and cause (seconds). **Comparison of pre-ACLF with early-stage ACLF. (DOC) [file pone.0064379.s001.doc]

**Table S1** Baseline characteristics of patients with ACLF and pre-ACLF

| **Variable** | **All patients**  **(*n* = 857)** | **pre-ACLF**  **(*n* = 190)** | **Early-stage ACLF**  **(*n* = 62)** | **Intermediate-stage ACLF**  **(*n* = 303)** | **Late-stage ACLF**  **(*n* = 302)** | ***p* value**** |
| --- | --- | --- | --- | --- | --- | --- |
| **Demographic** |  |  |  |  |  |  |
| Age (yr) | 46.1 ± 12.5 | 46.3 ± 13.2 | 39.7 ± 10.7 | 45.9 ± 12.3 | 47.3 ± 12.2 | ＜0.0001 |
| Sex (male/female) | 700/157 | 151/39 | 54/8 | 249/54 | 246/56 | 0.248 |
| **Primary cause** (%) |  |  |  |  |  |  |
| Hepatitis B | 70.3 | 67.9 | 79.0 | 70.3 | 69.9 | 0.095 |
| Hepatitis B plus alcohol abuse | 17.4 | 15.3 | 19.3 | 20.1 | 15.6 | 1.450 |
| Alcohol-related | 6.5 | 8.4 | 0.0 | 4.0 | 9.3 | 0.018 |
| Other* | 5.8 | 8.4 | 1.6 | 5.6 | 5.2 | 0.064 |
| **Acute cause (%)** |  |  |  |  |  |  |
| Hepatitis B | 55.3 | 49.5 | 67.7 | 57.8 | 54.0 | 0.012 |
| Hepatitis B plus other cause^##^ | 29.5 | 30.5 | 24.2 | 30.7 | 28.8 | 0.342 |
| Drug-related | 1.1 | 0.5 | 0.0 | 1.6 | 1.0 | 0.569 |
| Other^#^ | 14.1 | 19.5 | 8.1 | 9.9 | 16.2 | 0.036 |
| **Clinical** |  |  |  |  |  |  |
| Ascites (%) |  |  |  |  |  | ＜0.0001 |
| 0 None | 15.2 | 20.0 | 58.1 | 10.9 | 7.6 |  |
| 1 Detected only by ultrasonography | 51.9 | 56.3 | 37.1 | 52.8 | 51.3 |  |
| 2 Shifting dullness | 24.4 | 15.8 | 3.2 | 31.0 | 27.5 |  |
| 3 Tense ascites | 8.5 | 7.9 | 1.6 | 5.3 | 13.6 |  |
| Hepatic encephalophy (%) |  |  |  |  |  | 0.005 |
| Stage 0 | 68.2 | 85.8 | 98.4 | 74.9 | 44.1 |  |
| Stage Ⅰ | 15.5 | 9.5 | 1.6 | 17.5 | 20.2 |  |
| Stage Ⅱ | 8.4 | 2.1 | 0.0 | 7.3 | 15.2 |  |
| Stage Ⅲ | 4.2 | 0.5 | 0.0 | 0.0 | 11.6 |  |
| Stage Ⅳ | 3.7 | 2.1 | 0.0 | 0.3 | 8.9 |  |
| **Biochemical** |  |  |  |  |  |  |
| Albumin (g/dL) | 3.30±0.55 | 3.44±0.53 | 3.40±0.40 | 3.34±0.44 | 3.14±0.64 | 0.542 |
| Serum bilirubin (mg/dL) | 20.82±11.19 | 14.57±9.56 | 18.16±9.41 | 21.17±0.69 | 24.94±11.08 | 0.011 |
| Serum creatinine (mg/dL) | 1.05±0.94 | 0.87±0.49 | 0.79±0.35 | 0.91±0.77 | 1.36±1.26 | 0.238 |
| INR for prothrombin time | 2.20±1.15 | 1.43 ±0.19 | 1.73 ±0.56 | 1.94±0.71 | 2.63 ±1.58 | ＜0.0001 |
| Platelets (10^9/^L) | 101.82±59.31 | 103.00±83.00 | 110.00±74.25 | 94.00±64.00 | 80.50 ±72.25 | 0.978 |
| Triglycerides (mg/dL) | 92.76±72.43 | 114.70±94.11 | 88.57±58.90 | 78.83±57.57 | 61.11±55.80 | 0.044 |
| Sodium (mEq/L) | 135.49. ± 5.73 | 136.34 ± 4.68 | 137.48 ± 3.48 | 135.41 ± 5.22 | 134.62 ± 6.91 | 0.061 |
| **Scoring system** |  |  |  |  |  |  |
| CTP ^$^ | 10 ±1 | 10 ±1 | 10 ±1 | 10 ±1 | 10 ±1 | 0.489 |
| MELD ^&^ | 2.35 ± 0.90 | 1.77 ± 0.64 | 2.05 ± 0.54 | 2.25 ± 0.61 | 2.88 ± 0.98 | ＜0.0001 |
| MELD-Na ^$$^ | 2.76±4.88 | 1.86 ±2.04 | 2.18±0.96 | 2.61±4.52 | 4.31 ±8.54 | 0.157 |

Abbreviations: Abbreviation: ACLF, acute-to-chronic liver failure; INR, international normalized ratio; CTP, Child–Turcotte–Pugh scoring system; MELD, model for end-stage liver disease scoring system

Normal distribution continuous values were expressed as the mean±SD. Non-normal distribution continuous values were expressed as the median and interquartile range.

*Includes HBV combined with schistosome, HCV, alcohol combined with schistosome, autoimmune and cholestatic.

^#^Includes HGV, alcohol, HCV, autoimmune, surgery/trauma, and cholestatic.

^##^Includes HBV combined with drug, HBV combined with alcohol, HBV combined with HGV, HBV combined with HAV and HBV combined with surgery/trauma.

^$^ Based on hepatic encephalopathy, ascites, bilirubin, albumin, and prothrombin time (seconds).

^&^ Based on bilirubin, creatinine, INR and cause (seconds).

^$$^ Based on bilirubin, creatinine, INR, sodium and cause (seconds).

**Comparison of pre-ACLF with early-stage ACLF.
