# Supplementary material for: A Modified MELD Model for Chinese Pre-ACLF and ACLF Patients and It Reveals Poor Prognosis in Pre-ACLF Patients
Source: PLoS One. 2013 Jun 5;8(6):e64379. doi: 10.1371/journal.pone.0064379 (PMC3673980; doi:10.1371/journal.pone.0064379)
Supplement: Table S2 — Demographic, clinical, biochemical and hepatic-hemodynamic features in the training and validation cohorts. Abbreviations: Abbreviation: ACLF, acute-to-chronic liver failure; INR, international normalized ratio; CTP, Child–Turcotte–Pugh scoring system; MELD, model for end-stage liver disease scoring system NOTE: Normal distribution continuous values are expressed as the mean±SD. Non-normal distribution continuous values are expressed as the median and interquartile range. *Includes HBV combined with schistosome; HCV; alcohol combined with schistosome; autoimmune; and cholestatic. #Includes HGV, alcohol, HCV, autoimmune, surgery/trauma, and cholestatic. ##Includes HBV combined with drugs, HBV combined with alcohol, HBV combined with HGV, HBV combined with HAV, and HBV combined with surgery/trauma. $ Based on hepatic encephalopathy, ascites, bilirubin, albumin, and prothrombin time (seconds). & Based on bilirubin, creatinine, INR and cause (seconds). $$Based on bilirubin, creatinine, INR, sodium and cause (seconds). **Comparison of the training cohort with the validation cohort (DOC) [file pone.0064379.s002.doc]

**Table S2** Demographic, clinical, biochemical and hepatic-hemodynamic features in the training and validation cohorts

| **Variable** | **All patients**  **(n=857)** | **Training cohort**  **(n=758)** | **Validation cohort (n=99)** | ***P* value**** |
| --- | --- | --- | --- | --- |
| **Demographic** | | | | |
| Age (yr) (mean±SD) | 46.1±12.5 | 46.06±12.61 | 46.62±11.59 | 0.586 |
| Sex (male/female) | 700/157 | 618/140 | 82/17 | 0.754 |
| **Primary cause (%)** | | | | |
| Hepatitis B | 70.3 | 70.4 | 68.7 | 0.719 |
| Hepatitis B plus alcohol abuse | 17.4 | 17.0 | 20.2 | 0.432 |
| Alcohol-related | 6.5 | 6.6 | 6.0 | 0.839 |
| Other* | 5.8 | 6.0 | 5.1 | 0.724 |
| **Acute cause (%)** | | | | |
| Hepatitis B | 55.3 | 54.5 | 61.6 | 0.180 |
| Hepatitis B plus other cause^##^ | 29.5 | 30.2 | 24.2 | 0.221 |
| Drug-related | 1.1 | 1.1 | 1.1 | 0.967 |
| Other^#^ | 14.1 | 14.2 | 13.1 | 0.764 |
| **Clinical** | | | | |
| Ascites (%) |  |  |  | 0.614 |
| 0 None | 15.0 | 14.5 | 20.2 |  |
| 1 Detected only by ultrasonography | 52.0 | 52.6 | 46.5 |  |
| 2 Shifting dullness | 24.0 | 24.5 | 23.2 |  |
| 3 Tense ascites | 9.0 | 8.3 | 10.1 |  |
| Hepatic encephalophy(%) |  |  |  | 0.912 |
| Stage 0 | 68.0 | 66.8 | 78.8 |  |
| Stage Ⅰ | 16.0 | 16.8 | 6.1 |  |
| Stage Ⅱ | 8.0 | 8.6 | 7.1 |  |
| Stage Ⅲ | 4.0 | 4.0 | 6.1 |  |
| Stage Ⅳ | 4.0 | 4.0 | 2.1 |  |
| **Biochemical** (mean ± SD ) | | | | |
| Albumin (g/dL) | 3.30±0.55 | 3.30±0.56 | 3.25±0.44 | 0.345 |
| Serum bilirubin (mg/dL) | 20.82±11.19 | 20.89±11.08 | 20.29±12.03 | 0.617 |
| Serum creatinine (mg/dL) | 1.05±0.94 | 1.05±0.93 | 1.09±1.04 | 0.676 |
| INR for prothrombin time | 2.20±1.15 | 2.20±1.16 | 2.19±1.00 | 0.892 |
| Platelets (10^9^/L) | 101.82±59.31 | 101.77±59.02 | 102.21±61.82 | 0.945 |
| Triglycerides (mg/dL) | 92.76±72.43 | 90.77±73.36 | 108.01±63.14 | 0.026 |
| Sodium (mEq/L) | 135.49±5.73 | 135.68±5.64 | 134.06±6.19 | 0.008 |
| **Scoring system** (mean ± SD ) |  |  |  |  |
| CTP^$^ | 10 ± 1 | 10 ± 1 | 10 ± 1 | 0.811 |
| MELD^&^ | 2.35±0.90 | 2.35±0.89 | 2.32±1.00 | 0.459 |
| MELD-Na^$$^ | 2.76 ± 4.88 | 2.80 ± 4.68 | 2.46 ± 7.89 | 0.915 |

Abbreviations: Abbreviation: ACLF, acute-to-chronic liver failure; INR, international normalized ratio; CTP, Child–Turcotte–Pugh scoring system; MELD, model for end-stage liver disease scoring system

NOTE：Normal distribution continuous values are expressed as the mean±SD. Non-normal distribution continuous values are expressed as the median and interquartile range.

*Includes HBV combined with schistosome; HCV; alcohol combined with schistosome; autoimmune; and cholestatic.

^#^Includes HGV, alcohol, HCV, autoimmune, surgery/trauma, and cholestatic.

^##^Includes HBV combined with drugs, HBV combined with alcohol, HBV combined with HGV, HBV combined with HAV, and HBV combined with surgery/trauma.

^$^ Based on hepatic encephalopathy, ascites, bilirubin, albumin, and prothrombin time (seconds).

^&^ Based on bilirubin, creatinine, INR and cause (seconds).

^$$^Based on bilirubin, creatinine, INR, sodium and cause (seconds).

^**^Comparison of the training cohort with the validation cohort
